# Supplementary material for: Two-year recall for people with no diabetic retinopathy: a multi-ethnic population-based retrospective cohort study using real-world data to quantify the effect
Source: Br J Ophthalmol. 2023 Oct 24;107(12):1839–45. doi: 10.1136/bjo-2023-324097 (PMC10715554; doi:10.1136/bjo-2023-324097)
Supplement: Supplementary data [file bjo-2023-324097supp001.pdf]

Supplemental Table 2: Cumulative incidence rates of STDR per 100-person-years among PLD without diabetic retinopathy on 2 consecutive screening appointments, overall and by age groups, sex, ethnic group and IMD N= 82,782

Characteristic	Year 1	Year 2	Year 3	Year 4	Year 5	Year 9
**Overall**	0.20 (0.17 - 0.22)	0.33 (0.30 - 0.36)	0.40 (0.36 - 0.43)	0.44 (0.40 - 0.48)	0.45 (0.41 - 0.49)	0.51 (0.47 - 0.55)
**Age groups**						
Less than 45 years	0.19 (0.13 - 0.25)	0.35 (0.28 - 0.43)	0.44 (0.35 - 0.52)	0.48 (0.39 - 0.57)	0.52 (0.42 - 0.61)	0.67 (0.56 - 0.77)
45 to <55 years	0.18 (0.13 - 0.23)	0.31 (0.25 - 0.38)	0.36 (0.29 - 0.43)	0.41 (0.33 - 0.48)	0.42 (0.35 - 0.50)	0.49 (0.41 - 0.57)
55 to <65 years	0.17 (0.13 - 0.22)	0.27 (0.21 - 0.33)	0.32 (0.26 - 0.38)	0.35 (0.28 - 0.42)	0.35 (0.29 - 0.42)	0.39 (0.32 - 0.46)
65 years and over	0.24 (0.19 - 0.29)	0.38 (0.31 - 0.44)	0.47 (0.40 - 0.54)	0.51 (0.44 - 0.58)	0.51 (0.44 - 0.59)	0.53 (0.45 - 0.60)
**Sex**						
Female	0.22 (0.19 - 0.26)	0.35 (0.30 - 0.40)	0.44 (0.38 - 0.49)	0.48 (0.42 - 0.54)	0.48 (0.42 - 0.53)	0.54 (0.48 - 0.60)
Male	0.17 (0.14 - 0.21)	0.31 (0.27 - 0.35)	0.36 (0.31 - 0.41)	0.40 (0.35 - 0.45)	0.42 (0.37 - 0.47)	0.48 (0.43 - 0.54)
**Ethnicity**						
White	0.15 (0.12 - 0.19)	0.22 (0.18 - 0.27)	0.27 (0.22 - 0.32)	0.29 (0.24 - 0.35)	0.30 (0.25 - 0.35)	0.34 (0.29 - 0.40)
South Asian	0.21 (0.17 - 0.26)	0.37 (0.31 - 0.42)	0.43 (0.37 - 0.49)	0.47 (0.41 - 0.54)	0.48 (0.42 - 0.55)	0.55 (0.48 - 0.62)
Black	0.26 (0.18 - 0.33)	0.47 (0.37 - 0.57)	0.59 (0.49 - 0.70)	0.67 (0.56 - 0.79)	0.71 (0.59 - 0.83)	0.77 (0.65 - 0.90)
Any other Asian	0.23 (0.12 - 0.35)	0.29 (0.16 - 0.42)	0.39 (0.24 - 0.53)	0.44 (0.28 - 0.60)	0.43 (0.28 - 0.59)	0.48 (0.31 - 0.64)
Other	0.26 (0.09 - 0.43)	0.40 (0.18 - 0.61)	0.41 (0.19 - 0.63)	0.47 (0.23 - 0.70)	0.48 (0.25 - 0.72)	0.63 (0.36 - 0.90)
Mixed	0.20 (0.00 - 0.43)	0.60 (0.20 - 1.00)	0.69 (0.26 - 1.12)	0.61 (0.21 - 1.02)	0.54 (0.16 - 0.93)	0.67 (0.25 - 1.10)
Chinese	0.00 (0.00 - 0.00)	0.10 (0.00 - 0.31)	0.21 (0.00 - 0.52)	0.23 (0.00 - 0.56)	0.25 (0.00 - 0.60)	0.21 (0.00 - 0.52)
Unknown	0.16 (0.00 - 0.42)	0.67 (0.14 - 1.19)	0.59 (0.09 - 1.08)	0.55 (0.07 - 1.02)	0.52 (0.06 - 0.99)	0.50 (0.05 - 0.96)
**Type of diabetes**						
Type 2	0.20 (0.17 - 0.22)	0.32 (0.29 - 0.35)	0.39 (0.35 - 0.43)	0.43 (0.39 - 0.47)	0.44 (0.40 - 0.48)	0.50 (0.46 - 0.54)
Type 1	0.38 (0.16 - 0.60)	0.53 (0.27 - 0.79)	0.67 (0.38 - 0.96)	0.75 (0.44 - 1.05)	0.77 (0.46 - 1.09)	0.84 (0.52 - 1.17)
Other	0.00 (0.00 - 0.00)	0.42 (0.00 - 1.33)	0.96 (0.00 - 2.34)	0.82 (0.00 - 2.09)	0.75 (0.00 - 1.96)	0.86 (0.00 - 2.15)
**Index of Multiple Deprivation**						
1	0.16 (0.09 - 0.23)	0.25 (0.16 - 0.33)	0.36 (0.26 - 0.47)	0.43 (0.31 - 0.54)	0.45 (0.34 - 0.57)	0.48 (0.36 - 0.61)
2	0.22 (0.17 - 0.27)	0.37 (0.30 - 0.43)	0.43 (0.36 - 0.49)	0.47 (0.40 - 0.54)	0.48 (0.41 - 0.55)	0.54 (0.47 - 0.62)
3	0.17 (0.12 - 0.21)	0.32 (0.26 - 0.38)	0.40 (0.34 - 0.47)	0.43 (0.36 - 0.50)	0.43 (0.36 - 0.50)	0.50 (0.43 - 0.58)
4	0.25 (0.18 - 0.31)	0.34 (0.27 - 0.42)	0.38 (0.30 - 0.46)	0.42 (0.34 - 0.51)	0.43 (0.35 - 0.52)	0.49 (0.40 - 0.58)
5	0.17 (0.10 - 0.25)	0.31 (0.21 - 0.41)	0.36 (0.25 - 0.47)	0.41 (0.29 - 0.53)	0.42 (0.30 - 0.54)	0.48 (0.35 - 0.60)
